# Brief, Web-Based Interventions to Motivate Smokers With Schizophrenia: Randomized Controlled Trial

**DOI:** 10.2196/16524

**Published:** 2020-02-10

**Authors:** Mary F Brunette, Joelle C Ferron, Susan R McGurk, Jill M Williams, Amy Harrington, Timothy Devitt, Haiyi Xie

**Affiliations:** 1 Geisel School of Medicine at Dartmouth Dartmouth-Hitchcock Concord, NH United States; 2 Dartmouth-Hitchcock Concord, NH United States; 3 Boston University Boston, MA United States; 4 Rutgers New Brunswick, NJ United States; 5 University of Massachusetts Worcester, MA United States; 6 Thresholds, Inc Chicago, IL United States

**Keywords:** schizophrenia, smoking, tobacco, technology, digital, motivational interviewing, education, cognition

## Abstract

**Background:**

In-person motivational interventions increase engagement with evidence-based cessation treatments among smokers with schizophrenia, but access to such interventions can be limited because of workforce shortages and competing demands in mental health clinics. The use of digital technology to deliver interventions can increase access, but cognitive impairments in schizophrenia may impede the use of standard digital interventions. We developed an interactive, multimedia, digital motivational decision support system for smokers with schizophrenia (*Let’s Talk About Smoking*). We also digitalized a standard educational pamphlet from the National Cancer Institute (*NCI Education*). Both were tailored to reduce cognitive load during use.

**Objective:**

We conducted a randomized trial of *Let’s Talk About Smoking* versus *NCI Education* to test whether the interactive motivational intervention was more effective and more appealing than the static educational intervention for increasing use of smoking cessation treatment, quit attempts, and abstinence among smokers with schizophrenia, accounting for the level of cognitive functioning.

**Methods:**

Adult smokers with schizophrenia (n=162) were enrolled in the study from 2014 to 2015, randomly assigned to an intervention condition, and assessed in person at 3- and 6-month follow-ups. Interventions were delivered on a laptop computer in a single session. All participants had access to standard, community-delivered cessation treatments during follow-up. Multivariate models were used to evaluate outcomes.

**Results:**

Treatment initiation outcomes were not different between intervention conditions (27/84 [32%] for *Let’s Talk About Smoking* vs 36/78 [46%] for *NCI Education*; odds ratio [OR] 0.71 [95% CI 0.37-1.33]); 38.9% (63/162) of participants initiated treatment. Older age (OR 1.03 [95% CI 1.00-1.07]; *P*=.05), higher education (OR 1.21 [95% CI 1.04-1.41]; *P*=.03), and fewer positive symptoms (OR 0.87 [95% CI 0.80-0.96]; *P*=.01) predicted cessation treatment initiation, whereas level of cognition did not. The mean satisfaction and usability index score was higher for *Let’s Talk About Smoking* versus *NCI Education* (8.9 [SD 1.3] vs 8.3 [SD 2.1]; *t*_120.7_=2.0; *P*=.045). Quit attempts (25/84, 30% vs 36/78, 46%; estimate [Est]=−0.093, SE 0.48; *P*=.85) and abstinence (1/84, 1% vs 6/78, 7%; χ^2^_1_=3.4; *P*=.07) were not significantly different between intervention conditions. Cognitive functioning at baseline (Est=1.47, SE 0.47; *P*=.002) and use of any behavioral or medication cessation treatment (Est=1.43, SE 0.47; *P*=.003) predicted quit attempts with self-reported abstinence over the 6-month follow-up.

**Conclusions:**

The interactive, multimedia intervention was not more effective than the static, text-based intervention among smokers with schizophrenia. Both tailored digital interventions resulted in levels of treatment engagement and quit attempts that were similar to findings from previous studies of in-person interventions, confirming the potential role of digital interventions to educate and motivate smokers with schizophrenia to use cessation treatment and to quit smoking. These findings indicate that additional cessation treatment is needed after brief education or motivational interventions, and that cessation treatment should be adjusted for people with cognitive impairment.

**Trial Registration:**

ClinicalTrials.gov NCT02086162; https://clinicaltrials.gov/show/NCT02086162

## Introduction

### Background

Clinics serving people with schizophrenia aim to provide interventions for schizophrenia and the common comorbidities associated with this disease. Cigarette smoking, for example, is thrice more likely to occur in people with schizophrenia than in the general population [1,2] and leads to disparate morbidity from smoking-related diseases and early mortality [3]. However, workforce shortages are a challenge for community clinics in the United States [4,5] and interfere with the ability to provide the array of needed interventions for smoking. In addition, treatment providers experience competing demands and may lack clinical expertise for providing tobacco-related interventions [6,7]. Deploying digital tools to deliver behavioral interventions to patients is one way to improve the capacity for behavioral interventions.

People with schizophrenia and other severe mental illnesses are increasingly using digital technology and are interested in receiving health and mental health interventions via their devices [8-10]. However, people in this group typically have cognitive impairments and distracting symptoms that impede the use of standard digital tools that have complex design features and lower levels of usability [11-14]. To address this problem, we have designed digital tools with evidence-based content that can be easily used by people with cognitive impairments and easily implemented in treatment settings where smokers with schizophrenia receive services [15,16]. Other researchers are also beginning to design and pilot test smartphone apps for smoking cessation in this population [17-19].

One potential purpose for digital tools in clinics may be to educate and motivate a user for medical treatments. A growing body of literature indicates that cessation medications with behavioral interventions are safe among people with schizophrenia [20,21] and increase the probability of cessation [22-24]. Specifically, cognitive behavioral therapy, motivational counseling, and supportive counseling combined with nicotine replacement therapy, bupropion, or varenicline have been shown to improve cessation outcomes; behavioral interventions with varenicline have resulted in the highest rates of abstinence [20-24]. However, misperceptions about cessation treatment may impede their utilization [25-27]. Single-session [28,29] and multiple-session [30,31] in-person motivational and educational interventions for patients may overcome this problem, increasing treatment initiation and quit attempts among smokers with schizophrenia and other severe mental illnesses. Whether interventions delivered with digital technology can similarly increase cessation treatment initiation and quit attempts among people with schizophrenia has not yet been tested.

### Objectives

We conducted a randomized trial of a brief, interactive, multimedia intervention (*Let’s Talk About Smoking*) compared with a static, computerized version of an education pamphlet from the National Cancer Institute (NCI) among smokers with schizophrenia. Both interventions were tailored to reduce cognitive load on the user. We hypothesized that the rate of treatment initiation and cessation behaviors would be higher among participants assigned to *Let’s Talk about Smoking* than among those assigned to *NCI Education*. In addition, we hypothesized that the level of cognitive ability would moderate participants’ use of cessation treatment and ability to achieve abstinence.

## Methods

### Enrollment and Study Participants

**Figure 1:**
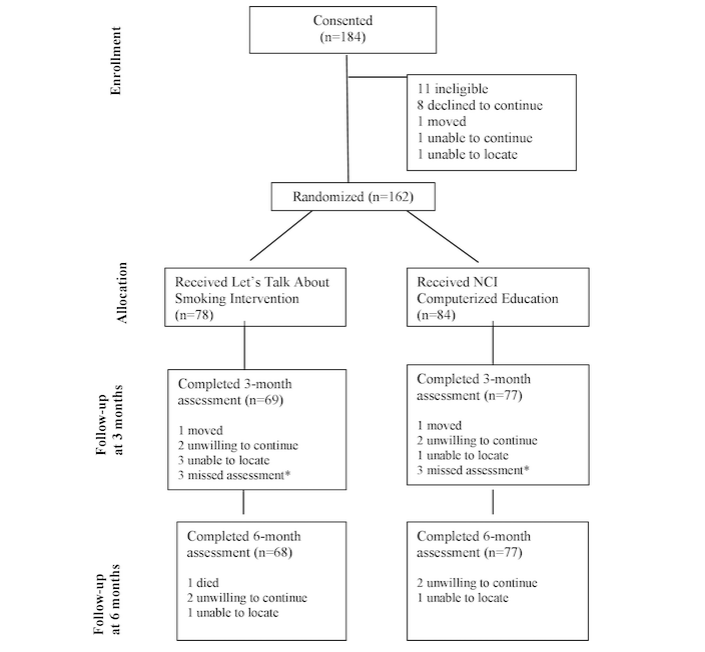
Study flow. *Participants missed 3-month visit but completed the 6-month visit. NCI: National Cancer Institute.

Potentially eligible smokers with schizophrenia were recruited via flyers in waiting rooms and by clinician invitation from mental health treatment programs in New Jersey, Massachusetts, and Illinois from 2014 to 2015. We enrolled English-speaking, daily smokers with schizophrenia spectrum disorders, aged 18 to 65 years, who were psychiatrically stable in outpatient treatment for mental illness (Brief Psychiatric Rating Scale (BPRS) score <70) [32] and who were willing and able to give informed consent. Smokers were excluded if they had recently (past month) used evidence-based smoking cessation treatment (indicating the participant was already motivated to use treatment), were pregnant or nursing, or had current untreated alcohol or drug dependence diagnoses. Computer experience was not required. As the intervention was designed to increase motivation for cessation, intention to quit smoking was not required. In total, 184 participants were consented and assessed for eligibility; 173 were eligible, 162 were randomized and received study interventions, and 145 (89.5% of those randomized) completed the 6-month follow-up (see Figure 1 for participant flow).

### Study Procedures

After obtaining informed consent through reading the consent form aloud and answering questions, research staff conducted baseline assessments in 2 in-person meetings, with neurocognitive assessments obtained at the second meeting to reduce fatigue. Within 2 weeks of consent, eligible participants were randomized 1:1 to receive one of the interventions using computer-generated random order lists in blocks of 8, stratified by study site, with study participant allocation provided via preprepared, individual envelopes that were unsealed by research staff at the time the participant arrived for the intervention visit. Participants were not informed of the details of the study hypothesis and did not know which comparator was hypothesized to outperform the other.

Using a standard protocol, research staff oriented participants to their assigned intervention, which was provided in a clinic office on a laptop computer with a mouse. They provided brief training, coaching, and assistance if needed. After completing either intervention, participants completed a computerized satisfaction questionnaire (to reduce social desirability bias) and received referral information to locally available cessation treatment (cessation medications and cessation counseling) by clinicians who were trained in providing evidence-based cessation treatment to people with serious mental illnesses (SMIs). At 3 and 6 months, research interviewers who were blinded to intervention assignment assessed participants in person for the use of verifiable cessation treatment (main outcome), smoking characteristics, self-reported quit attempts (days of abstinence), and biologically verified abstinence (secondary outcomes; see Measures section). Research staff provided participants US $50 on completion of each assessment visit. Data quality was monitored throughout the study by the first author, the research data team, and a Data Safety and Monitoring Board. The study was reviewed and monitored by the Dartmouth Committee for the Protection of Human Subjects and the Institutional Review Boards of research sites.

### Intervention Conditions

#### Web-Based Motivational Intervention

*Let’s Talk About Smoking* is a Web-based intervention tailored for smokers with severe mental illnesses and designed to increase motivation to quit smoking using evidence-based treatment. The development of the intervention’s content and interface involved extensive input from the intended users and has been described previously [15]. The program is linear, modularized, and interactive, taking 30 to 90 min to complete. Users choose a video host who identifies him/herself as an ex-smoker with mental illness and guides users through modules, each with assessments and exercises used in motivational interviewing and health decision aid systems [33,34]. In module 1 (assessment/feedback), users respond to questions and receive personalized feedback about the personal, financial, and health impact of smoking. In module 2 (quit intention), change decisions are facilitated by cessation treatment information and exercises, including creation of a personalized pros and cons list. Module 3 (education about cessation treatments, feedback, and referral) provides selectable quit story videos as well as text and video information about cessation treatments, including the benefits of combined behavioral counseling with pharmacotherapy. A personalized report highlights the desire to quit, treatment choices, and referral information. The developers and their institutions were listed at the end of the intervention.

By developing the intervention interface and content with iterative user feedback, we ensured that the intervention was easy to use among people with the symptoms and cognitive impairments associated with psychotic disorders [15]. We previously showed that the decision support system was similarly effective among smokers with high and low levels of education, cognitive function, and symptom distress [35]. The intervention content remained constant during the trial.

#### Computerized National Cancer Institute Patient Education

Participants assigned to *NCI Education* received a computerized version of the NCI patient educational handout [36], which provides information about risk factors and protective factors for cancer and other smoking-related diseases, quitting smoking as a prevention factor, and smoking cessation treatments (both counseling and drug treatments, including nicotine replacement therapy, bupropion, and varenicline). This static intervention was delivered by a laptop computer in a format similar to *Let’s Talk About Smoking*: large black font on a white background with no distracting images; one concept per page in a short paragraph or bulleted sentences. Automated audio, which read the content to users, could be turned on if the user wished. The publisher of the pamphlet, the NCI, was named as sponsor of the pamphlet in standard text in the beginning and at the end of the intervention.

### Measures

#### Demographics, History, and Diagnosis

Demographics and smoking history were assessed with a structured, in-person interview. Physician-completed Diagnostic and Statistical Manual, Fourth Edition, Text Revision, psychiatric and substance use disorder diagnoses were obtained from clinic chart review.

#### Mental Health Symptoms

Trained research staff assessed psychiatric symptom severity at baseline with the BPRS [32], a widely used symptom scale for symptoms of mental illness. The scale includes five subscales that measure positive psychosis symptoms, negative psychosis symptoms, depression, disorganized symptoms, and activation [37].

#### Smoking Characteristics

Research staff assessed all participants for the level of nicotine dependence with the Fagerström Test for nicotine dependence at baseline and at 3 and 6 months [38-40].

#### Motivation for Cessation and Treatment

We assessed participants for their stage of change for quitting smoking with the single question, “Are you seriously thinking about quitting?” [41]. We also assessed attitudes about using cessation treatment with an adapted Treatment Motivation Scale-Revised, a 23-item scale assessing attitudes about using treatment based on self-determination theory [42]. This scale has five additive subscales that assess perceptions of reasons for treatment, including external motivation (range 4-20), introjected motivation (range 2-10), intrinsic motivation (range 7-35), lack of confidence in using treatment (range 4-20), and relatedness in treatment (range 7-35) [43].

#### Primary Outcome—Confirmed Use of Smoking Cessation Treatment and Quit Attempts

Blinded assessors completed a structured interview to assess all self-reported use of cessation treatment (including nicotine replacement therapy) at any time during each past 3-month period. The use of cessation treatment was confirmed via clinic record review, clinician confirmation, and viewing medications and nicotine replacement at the assessment. The use of cessation treatment and quit attempts were expected to directly result from the use of the study interventions.

#### Secondary Outcome—Abstinence

At the follow-up assessment visits, the self-reported, past week of abstinence from smoking was verified with expired carbon monoxide less than 9 ppm (Smokelyzer Breath Carbon Monoxide Monitor; Bedfont Scientific) [44,45]. In addition, any self-reported quit attempts with abstinence during the treatment period were captured with the Timeline Follow-Back method [46-48]. With this method, trained research staff assessed participants for the amount of smoking and other tobacco product use each day, going back week-by-week over the past 3 months using a calendar to cue memories of smoking and abstinence. The Timeline Follow-Back method has been shown to be reliable and valid in the general population [48] and in people with severe mental illnesses [49]. Abstinence was identified as a secondary outcome that would rely on the use of additional cessation medication and behavioral cessation treatment.

#### Intervention Satisfaction, Usability, and Likeability

Participants completed the Perceived Usefulness and Ease of Use Scale, an adapted 15-item semiqualitative instrument [50] to obtain perceptions of usability and satisfaction with the intervention.

#### Cognition

We assessed cognition at baseline with a battery comprised of the following 6 standard neuropsychological tests that measure cognitive functions typically impaired in schizophrenia and thought to be important for engagement and success in smoking cessation treatment (Multimedia Appendix 1). We assessed sustained attention (Continuous Performance Test, dependent variable: d’) [51], verbal learning (Hopkins Verbal Learning Test; dependent variable: total recall trials 1-3; *t* score from mean of the three trials)[52,53], processing speed (Trail Making Test Part A; dependent variable: seconds to completion) [54], and, because of the likelihood of important relationships of nicotine abstinence and the prefrontal cortex [55-57], we assessed cognitive flexibility (Trail Making Test Part B: dependent variable: seconds to completion) and inhibitory control (Delis-Kaplan Executive Functioning System Color-Word Interference Test; dependent variable: seconds to completion on word reading, color reading, and color-word interference trials) [58]. The mean of a participant’s normative scores was used as a composite cognition score. Composite scores were not computed for people who had one or more missing test score.

We also measured word recognition at baseline, calculated from a demographically based index of premorbid intelligence (fourth edition of the Wide Range Achievement Test Reading subtest) [59]. Performance on this test is relatively preserved in people with schizophrenia [60], providing an index of premorbid intellectual function.

### Statistical Analyses

We used chi-square tests and *t* tests to assess between-group differences at baseline. We then assessed dichotomous outcomes between intervention groups with logistic regressions (eg, treatment use) [61]. For count outcome variables with a high proportion of zeros and positive skewness (eg, days of abstinence), negative binomial models were used. Modeling began with bivariates and progressed to multivariates using variables providing *P*<.10 in bivariate models, adjusting for gender and years of education. In the multivariate model predicting any abstinence, the total mean cognitive battery score was used to avoid collinearity among the cognitive function scores. Missing observations for the primary outcome, cessation treatment utilization, were set as missing. Missing observations for the secondary outcome, abstinence, were set as smoking (nonabstinent). Analyses were conducted with SAS version 9.4 (SAS Institute, Cary, North Carolina).

## Results

### Overview

Participants are described in Multimedia Appendix 1. The group included 162 smokers with schizophrenia, with a mean age of 45.91 years (SD 11.32). Two-thirds were male (108/162, 66.7%), more than half identified as black (86/162, 53%). The group was moderately symptomatic (BPRS mean score 41.06, SD 11.11) and reported a mean of 11.12 (SD 13.69) hospitalizations for psychiatric treatment over their lifetimes, demonstrating long-term severe mental illness. Participants smoked an average of 14.56 cigarettes per day (SD 10.59). A low proportion (8.02%) of participants were motivated to quit smoking, and the level of motivation to use cessation treatment was generally low, and it was lowest in perceived external sources of motivation. The group demonstrated moderate cognitive impairments, as expected among people with schizophrenia. Characteristics were not significantly different between participants in the *Let’s Talk About Smoking* and *NCI Education* conditions.

### Primary Outcome

As shown in Table 1, more than one-third (63/162, 38.9%) of all participants used any verifiable cessation treatment during the 6-month follow-up period, and cessation treatment use was not different between intervention groups (27/84, 32.1% of *Let’s Talk About Smoking* vs 36/78, 46.2% *NCI Education*; odds ratio [OR] 0.71 [0.37-1.33]; *P*=.28).

**Table 1:** Confirmed cessation behaviors over 6-month follow-up.

Cessation behaviors		Total sample (N=162)	Let’s Talk About Smoking (N=84)	National Cancer Institute Education (N=78)
**Verified use of cessation treatment, n (%)**				
	Met with doctor to discuss cessation	72 (44.4)	37 (44)	35 (45)
	Nicotine replacement therapy	34 (20.9)	18 (21)	16 (21)
	Bupropion	7 (4.3)	6 (8)	1 (1)
	Varenicline	3 (1.9)	0 (0)	3 (4)
	Individual cessation counseling	35 (21.6)	16 (19)	19 (24)
	Group cessation counseling	15 (9.3)	7 (8)	8 (10)
	Cessation counseling and medication	21 (13.0)	12 (14)	9 (11)
	Started any treatment	63 (38.9)	27 (32)	36 (46)
**Self-reported or verified use of cessation treatment, n (%)**				
	Met with doctor to discuss cessation	92 (56.8)	44 (56)	48 (57)
	Nicotine replacement therapy	52 (32.1)	24 (31)	28 (33)
	Bupropion	13 (8.0)	8 (10)	5 (6)
	Varenicline	15 (9.3)	7 (9)	8 (10)
	Individual cessation counseling	52 (32.1)	25 (32)	27 (32)
	Group cessation counseling	25 (15.4)	10 (13)	15 (18)
	Cessation counseling and medication	35 (21.6)	17 (22)	18 (231)
	Started any treatment	82 (50.6)	34 (44)	48 (57)
**Abstinence outcomes, n (%)**				
	Verified abstinence at 6 months^a^	7 (4.3)	1 (1)	6 (8)
	Any quit attempt with ≥1 day abstinence^b^	61 (37.2)	25 (30)	36 (46)
	Any quit attempt with ≥7 days abstinence^b^	24 (14.8)	13 (15)	11 (14)

^a^Calculated from randomized sample.

^b^Calculated from follow-up sample.

Table 1 shows the number of participants who used each type of cessation treatment. Of the 63 participants who used any type of cessation treatment, some individuals used several types of medications, and some used group and individual behavioral cessation counseling. Of 162 participants, 21 (13.0%) had used at least one type of any verified cessation medication, 21 (13.0%) had used at least one type of any verified behavioral intervention, and the same number had used the recommended combination of both a behavioral and a medication intervention (21/162, 13.0%; these summary numbers are not shown in Table 1). A larger number of participants self-reported the use of treatment or had verified the use of treatment (also shown in Table 1). In bivariate logistic models, any verified treatment initiation was significantly predicted by older age (OR 1.03 [95% CI 1.00-1.06]; *P*=.05), higher levels of education (OR 1.18 [95% CI 1.02-1.37]; *P*=.02), and lower positive symptom scale scores (OR 0.87 [95% CI 0.79-0.95); *P*<.001). In the full multivariate model predicting cessation treatment utilization, older age, higher education, and lower level of positive symptoms, scores remained significant predictors of treatment initiation (see Table 2).

**Table 2:** Predictors of treatment initiation after brief interventions.

Demographic and smoking characteristics			Univariate models^a^			Multivariate models^a^		
			OR^b^	95% CI	*P* value	OR	95% CI	*P* value
Gender			1.09	0.57-2.06	.80	N/A^c^	N/A	N/A
Age			1.03	1.00-1.06	.05	1.03	1.00-1.07	.05
Education			1.18	1.02-1.37	.02	1.21	1.04-1.41	.02
Fagerström Score			1.03	0.88-1.21	.74	N/A	N/A	N/A
Cigarettes per day			1.00	0.97-1.03	.89	N/A	N/A	N/A
**Cognitive function**								
	TM^d^ A time		1.00	0.99-1.01	.80	N/A	N/A	N/A
	TM B time		1.00	1.00-1.00	.30	N/A	N/A	N/A
	Color time		0.99	0.96-1.02	.53	N/A	N/A	N/A
	Word time		1.00	0.96-1.03	.83	N/A	N/A	N/A
	Interfere T		0.99	0.98-1.01	.24	N/A	N/A	N/A
	Hopkins Verbal Learning Test		1.06	0.87-1.28	.56	N/A	N/A	N/A
	Continuous performance test		0.89	0.76-1.03	.12	N/A	N/A	N/A
	Cognition_Total_^e^		0.97	0.61-1.55	.91	N/A	N/A	N/A
**Symptoms**								
	**BPRS^f^ subscales**							
		Positive	0.87	0.79-0.95	<.001	0.87	0.80-0.96	.01
		Negative	0.95	0.81-1.11	.52	N/A	N/A	N/A
		Activation	0.97	0.84-1.12	.69	N/A	N/A	N/A
		Depression	0.94	0.85-1.04	.24	N/A	N/A	N/A
		Disorganized	0.95	0.83-1.08	.42	N/A	N/A	N/A
	BPRS total score		0.97	0.94-1.00	.06	N/A	N/A	N/A
	PANAS^g^ positive		1.01	0.98-1.05	.50	N/A	N/A	N/A
	PANAS negative		1.01	0.98-1.05	.47	N/A	N/A	N/A
**Intervention**								
	Intervention group		0.71	0.37-1.33	.28	0.65	0.33-1.31	.23

^a^Logistic regression models.

^b^OR: odds ratio.

^c^N/A: not applicable.

^d^TM: trial making.

^e^Only total cognition score was included in multivariate model.

^f^BPRS: Brief Psychiatric Rating Scale.

^g^PANAS: Positive and Negative Affect Schedule.

### Secondary Outcome

Although more than one-third of participants (61/162, 37.7%) reported that they had tried to quit and 24 participants (24/162, 14.8%) reported at least 7 days of self-reported abstinence over the follow-up period, only 4.3% (7/162) of participants had biologically verified 7-day point prevalence abstinence at the 6-month assessment (1/78, 1%, in *Let’s Talk About Smoking* vs 6/84, 7%, in *NCI Education*; χ^2^_1_=3.4; *P*=.07). Quit attempts and abstinence were not significantly different between intervention groups. In bivariate models predicting any self-reported abstinence during the follow-up period, greater level of education (beta=.214; SE 0.11; *P*=.04), greater positive affect (beta=.055; SE 0.03; *P*=.05), better overall cognitive functioning (composite score; beta=1.293; SE 0.42; *P*=.0002), and use of any cessation treatment (beta=1.112; SE 0.48; *P*=.02) significantly predicted abstinence (see Table 3). Better performance on most of the individual cognition scale scores also predicted self-reported abstinence. In adjusted multivariate models predicting days of abstinence, greater cognitive ability composite score and engagement in cessation treatment significantly predicted days of abstinence (see Table 3).

**Table 3:** Predictors of abstinence after brief interventions.

Demographic and clinical characteristics			Univariate models^a^			Multivariate models^a^		
			Est^b^	SE	*P* value	Est	SE	*P* value
Gender			−0.155	0.51	.80	N/A^c^	N/A	N/A
Age			0.001	0.02	.96	N/A	N/A	N/A
Education			0.214	0.11	.04	0.110	0.09	.22
Fagerström			−0.093	0.13	.50	N/A	N/A	N/A
Cigarettes per day			−0.025	0.02	.20	N/A	N/A	N/A
**Cognitive function**								
	TM^d^ A time		−0.024	0.01	.02	N/A	N/A	N/A
	TM B time		−0.003	0.00	.06	N/A	N/A	N/A
	Color time		−0.056	0.02	.005	N/A	N/A	N/A
	Word time		−0.056	0.03	.04	N/A	N/A	N/A
	Interfere T		−0.020	0.01	.05	N/A	N/A	N/A
	Hopkins Verbal Learning Test		0.166	0.19	.40	N/A	N/A	N/A
	Continuous performance test		0.256	0.11	.02	N/A	N/A	N/A
	Cognition_Total_^e^		1.293	0.42	.002	1.471	0.47	.002
**Symptoms**								
	**BPRS^f^ subscales**							
		Positive	−0.011	0.07	.90	N/A	N/A	N/A
		Negative	−0.091	0.13	.50	N/A	N/A	N/A
		Activation	0.031	0.09	.70	N/A	N/A	N/A
		Depression	0.057	0.08	.50	N/A	N/A	N/A
		Disorganized	−0.016	0.10	.90	N/A	N/A	N/A
	BPRS total score		0.015	0.02	.50	N/A	N/A	N/A
	PANAS^g^ positive		0.055	0.03	.05	0.004	0.03	.91
	PANAS negative		0.009	0.03	.80	N/A	N/A	N/A
**Intervention and cessation treatment**								
	Intervention group		−0.093	0.48	.85	0.155	0.46	.74
	Engaged in cessation treatment		1.112	0.48	.02	1.427	0.47	.003

^a^Negative binomial models.

^b^Est: estimate.

^c^N/A: not applicable.

^d^TM: trial making.

^e^Only total cognition score was included in multivariate model.

^f^BPRS: Brief Psychiatric Rating Scale.

^g^PANAS: Positive and Negative Affect Scale.

### Intervention Usability and Satisfaction

Usability and satisfaction mean summary index scores were significantly higher among participants assigned to *Let’s Talk About Smoking* compared with those assigned to *NCI Education* (8.9 [SD 1.3] vs 8.3 [SD 2.1]; *t*_120.7_=2.0; *P*=.045). Most participants (95.38% of *Let’s Talk About Smoking* users vs 83.1% of *NCI Education* users) reported that they were satisfied or very satisfied with the intervention. All participants completed the intervention to which they were assigned; no adverse events were reported during the use of the interventions. Approximately 97% of both groups said they would recommend their respective intervention to a friend.

## Discussion

### Principal Findings

To our knowledge, this is the first randomized trial testing an interactive, multimedia digital motivational intervention to a static digital educational intervention for motivating smokers with schizophrenia to try to quit smoking using evidence-based cessation treatment. Contrary to our hypothesis, smokers with schizophrenia assigned to the interactive intervention were not more likely to initiate cessation treatment. However, these brief, digital interventions led to rates of treatment engagement consistent with studies of earlier versions of *Let’s Talk About Smoking* [62-64] and consistent with in-person motivational interviewing, in which 28% to 32.7% of smokers with schizophrenia and bipolar disorder attended an initial treatment appointment [28,29]. Similar to other studies of digital tools for people with schizophrenia and other SMIs [65,66], this study suggests that carefully designed, automated, digital interventions are feasible and acceptable among people with schizophrenia. Such tools could be used to engage smokers with schizophrenia into quit attempts using evidence-based smoking cessation treatment, potentially reducing demands on clinicians and clinics serving this population.

Although both interventions were rated highly, the interactive, multimedia intervention was significantly more appealing than the static educational intervention. In a previous study, young adults with SMI rated the video content of *Let’s Talk About Smoking* the highest among the various types of content [67]. In nonstudy environments, future uptake of digital interventions might be most successful with a multimedia approach, including video compared with text-only interventions such as *NCI Education*.

The computerized *NCI Education* performed numerically but not statistically significantly better than *Let’s Talk About Smoking* in this study, and numerically better than in a previous study of a paper pamphlet (15% initiated treatment) [63] and in-person interactive education (20.4% initiated treatment) [28]. The outcomes with *NCI Education* were likely facilitated by design features that facilitated comprehension and cognitive processing, including high contrast text with large font; audio in addition to text; presentation of a single concept per page; and sequential, linear formatting of the information. All these design features were also used in the interactive, multimedia intervention. Although video media is very appealing to users, this study indicates that it does not provide an advantage over text-only interventions within a research context.

In this study, the use of any behavioral and pharmacologic cessation treatment following the study interventions significantly predicted abstinence, confirming that motivational and educational interventions should be followed by combined pharmacologic and behavioral interventions [22,24] in order for smokers with schizophrenia to achieve abstinence. Rates of biologically verified abstinence were consistent with what would be expected, given the types of cessation treatment used by the 61 participants who initiated treatment (7/61, 11% of abstinence). For example, 6 months after initiating treatment with a 3-month trial of bupropion, 4% of smokers with schizophrenia were abstinent [68]. Providing more Web-based motivational content for cessation and treatment utilization over repeated sessions and educating the clinicians to encourage and provide combined behavioral interventions and pharmacotherapy may improve utilization of the most effective combinations of treatments. Many community mental health centers do not include cessation treatment in their service array; thus, external services may be needed.

Achieving abstinence is a challenging task requiring multiple cognitive functions. Better performance on our battery of tests assessing aspects of prefrontal functioning, such as cognitive flexibility and inhibitory control, significantly predicted abstinence over the 6-month follow-up, although participants initiated treatment and attempted to quit smoking regardless of the level of cognitive functioning, similar to our previous study [35]. Consistent with the abstinence finding here, previous studies have shown that lower scores on attention [55,69,70], information processing [70], and inhibitory control [71] were associated with worse cessation outcomes in smokers with schizophrenia, although not all studies are consistent with these findings. Although we did not measure working memory, other studies have also shown that working memory was associated with abstinence outcomes [70,71]. Attention, concentration, memory, working memory, and inhibitory control are arguably needed to learn smoking cessation skills and to use them while inhibiting the urge to smoke. Cognitive remediation interventions have been shown to improve cognition and functional outcomes among people with SMI who are receiving psychosocial interventions [72]. One promising initial study of cognitive remediation added to addiction treatment enhanced substance abuse outcomes among people with schizophrenia [73]. Cognitive remediation delivered with smoking cessation treatment has not been tested among smokers with schizophrenia.

These results among middle-aged smokers with schizophrenia contrast with our previous work among young adults with SMIs [74]. In young adult smokers with SMI, the use of a similar digital intervention resulted in greater numbers of quit attempts and a greater proportion of people with biologically verified abstinence but less use of cessation treatment in the 3 months following the intervention [67].

Several study limitations should be mentioned. First, this study used an active, computerized control condition; thus, we were unable to determine the level of advantage these interventions provide over usual care, such as doctor’s advice. Second, we were not able to obtain detailed information about the frequency and intensity of the community-delivered cessation medication and behavioral interventions, which would have facilitated a better understanding of our secondary abstinence outcome. Finally, study participants were recruited from three large community clinics in three states and included smokers with schizophrenia from several racial and ethnic groups, yet they may not be representative of all smokers with schizophrenia in the United States or other countries.

### Conclusions

The interactive, multimedia, digital intervention was not more effective than a static digital intervention tailored to reduce cognitive load among smokers with schizophrenia. Both brief digital interventions garnered results similar to those found in previous studies of in-person motivational interventions among smokers with SMIs. Technology-delivered tobacco treatments have the promise to expand access in this high need population with high rates of smoking in clinics with longstanding workforce challenges but must be developed with user input and tested for efficacy, address data safety and privacy, and eventually integrate with electronic medical records and data systems [75]. Technology-delivered tobacco treatments could provide brief or long-term cessation skills training and cessation support, which could augment or replace in-person interventions for this population, as has been shown to be effective for the treatment of addiction in the general population [76]. Further research is warranted to evaluate efficacy and implementation strategies for digital interventions for smokers with schizophrenia and other SMIs.

## Appendix

Multimedia Appendix 1 Baseline demographics and characteristics of 162 study participants.

Multimedia Appendix 2 CONSORT-EHEALTH checklist (V 1.6.2).

## Abbreviations

BPRS: Brief Psychiatric Rating Scale
Est: estimate
NCI: National Cancer Institute
OR: odds ratio
SMI: serious mental illness
