# Supplementary material for: Brief, Web-Based Interventions to Motivate Smokers With Schizophrenia: Randomized Controlled Trial
Source: JMIR Ment Health. 2020 Feb 10;7(2):e16524. doi: 10.2196/16524 (PMC7055792; doi:10.2196/16524)
Supplement: Multimedia Appendix 1 [file mental_v7i2e16524_app1.docx]

MultiMedia Appendix

Table 1. Baseline demographics and characteristics of 162 study participants.

| Participant characteristics | | | Total (N=162) | Let’s Talk About Smoking (N=78) | National Cancer Institute Education (N=84) |
| --- | --- | --- | --- | --- | --- |
| Age (years), mean (SD) | | | 45.91 (11.3) | 47.63 (11.3) | 44.32 (11.2) |
| Years of education, mean (SD) | | | 11.88 (2.4) | 11.56 (2.1) | 12.17 (2.5) |
| **Sex, n (%)** | | | | | |
|  |  | Male | 108 (66.7) | 49 (62.8) | 59 (70.2) |
| **Race, n (%)** | | | | | |
|  |  | White | 47 (29.0) | 20 (25.6) | 27 (32.1) |
|  |  | Black | 86 (53.1) | 44 (56.4) | 42 (50.0) |
|  |  | Mixed and other | 29 (17.9) | 14 (18.0) | 15 (17.9) |
| **Ethnicity, n (%)** | | | | | |
|  |  | Hispanic | 21 (13.0) | 12 (15.4) | 9 (10.7) |
| **Marital status, n (%)** | | | | | |
|  |  | Single | 125 (77.2) | 62 (79.5) | 63 (75.0) |
| Employed, n (%) | | | 12 (7.4) | 4 (5.1) | 8 (9.5) |
| **Tobacco use characteristics** | | | | | |
|  | Cigarettes per day, mean (SD) | | 14.56 (10.5) | 14.48 (9.6) | 14.64 (11.3) |
|  | Fagerström dependence score, mean (SD) | | 5.16 (2.0) | 5.03 (2.2) | 5.29 (1.8) |
|  | Breath carbon monoxide, parts per million (SD) | | 27.16 (19.8) | 26.21 (18.2) | 28.06 (21.2) |
|  | **Participants with daily use of tobacco product^a^, n (%)** | | | | |
|  |  | Prerolled^b^ | 151 (93.1) | 76 (97.4) | 75 (89.3) |
|  |  | Rolls own | 31 (19.1) | 11 (14.1) | 20 (23.8) |
|  |  | Both | 20 (12.4) | 9 (11.5) | 11 (13.1) |
|  |  | Menthol | 128 (79.0) | 61 (78.2) | 67 (79.8) |
|  |  | Mini cigars (pseudo cigarettes) | 42 (25.9) | 20 (25.6) | 22 (26.2) |
|  |  | Cigarillos | 10 (6.2) | 5 (6.41) | 5 (6.0) |
|  |  | Electronic cigarette | 7 (4.3) | 4 (5.1) | 3 (3.6) |
|  | **Participants with motivational characteristic, n (%)** | | | | |
|  |  | Tried to quit in the past 3 months | 43 (26.5) | 19 (24.4) | 24 (28.6) |
|  |  | Previous use of nicotine replacement therapy | 19.00 | 7 (9.0) | 12 (14.3) |
|  |  | Previous use of other cessation medications | 1.00 | 0 (0) | 1 (1.2) |
|  | **Stage of change, n (%)** | | | | |
|  |  | Trying to quit now | 13 (8.0) | 5 (6.4) | 8 (9.5) |
|  |  | Thinking of quitting in the next month | 37 (22.8) | 21 (26.9) | 16 (19.1) |
|  |  | Thinking of quitting but not in the next month | 49 (30.3) | 25 (32.1) | 24 (28.6) |
|  |  | Not thinking of quitting | 63 (38.9) | 27 (34.6) | 36 (42.9) |
|  | **Treatment Motivation Questionnaire, subscale scores, mean (SD)** | | | | |
|  |  | Lack of confidence in treatment (4-20) | 12.76 (4.6) | 12.86 (4.8) | 12.67 (4.3) |
|  |  | External motivation (4-20) | 7.66 (3.2) | 7.80 (3.3) | 7.52 (3.2) |
|  |  | Introjected motivation (2-10) | 3.76 (2.1) | 3.91 (2.2) | 3.6 (2.0) |
|  |  | Intrinsic motivation (7-35) | 17.86 (5.7) | 18.10 (16.7) | 17.63 (5.3) |
|  |  | Relatedness in treatment (7-35) | 19.88 (7.0) | 19.87 (7.1) | 19.88 (6.9) |
| **Clinical characteristics** | | | | | |
|  | Lifetime hospitalizations, mean (SD) | | 11.12 (13.7) | 9.15 (9.7) | 12.95 (16.4) |
|  | Wide Range Achievement Test reading, mean (SD) | | 49.92 (10.4) | 48.81 (10.0) | 50.98 (10.8) |
|  | Brief Psychiatric Rating Scale, total score, mean (SD) | | 41.06 (11.1) | 41.79 (12.0) | 40.38 (10.3) |
|  | Positive symptoms, mean (SD) | | 12.12 (5.4) | 12.19 (5.5) | 12.05 (5.3) |
|  | Negative symptoms, mean (SD) | | 9.83 (3.7) | 10.06 (3.9) | 9.61 (3.6) |
|  | Agitation symptoms, mean (SD) | | 8.82 (3.2) | 8.91 (3.6) | 8.74 (2.7) |
|  | Depression symptoms, mean (SD) | | 10.29 (4.1) | 10.62 (4.4) | 9.99 (3.9) |
|  | PANAS^c^: positive score, mean (SD) | | 22.33 (8.5) | 22.36 (8.7) | 22.31 (8.5) |
|  | PANAS: negative score, mean (SD) | | 12.05 (9.3) | 12.04 (9.1) | 12.06 (9.5) |
|  | Alcohol use past 6 months, n (%) | | 67 (41.4) | 34 (43.6) | 33 (39.3) |
|  | Drug use past 6 months, n (%) | | 29 (17.9) | 15 (19.2) | 14 (16.7) |
| **Neuropsychiatric test, mean scores (SD)** | | | | | |
|  | **TM^d^** | | | | |
|  |  | TM A time (seconds) | 49.23 (29.2) | 49.01 (24.3) | 49.28 (33.8) |
|  |  | TM B time (seconds) | 219.01 (162.1) | 237.7 (167.6) | 194.89 (154.2) |
|  | **Hopkins Verbal Learning Test** | | | | |
|  |  | Trial 1 | 4.87 (1.7) | 5.01 (1.6) | 4.80 (1.8) |
|  |  | Trial 2 | 6.59 (2.0) | 6.68 (1.7) | 6.62 (2.2) |
|  |  | Trial 3 | 7.43 (2.1) | 7.54 (2.10) | 7.45 (2.1) |
|  |  | Total trial *t* score | 34.38 (7.2) | 34.79 (6.94) | 34.45 (7.5) |
|  | **Stroop** | | | | |
|  |  | Time color reading (seconds) | 40.29 (11.5) | 39.22 (9.7) | 40.54 (11.8) |
|  |  | Time word reading (seconds) | 29.15 (9.8) | 28.28 (6.8) | 29.05 (10.3) |
|  |  | Time interferences (seconds) | 76.91 (25.3) | 78.05 (24.4) | 74.89 (24.3) |
|  | **Continuous performance task (CPT)** | | | | |
|  |  | CPT D’^e^ | 1.83 (0.7) | 1.85 (0.8) | 1.85 (0.7) |
|  | **Cognitive composite *t* score** | | 32.89 (10.9) | 33.39 (11.2) | 32.77 (10.6) |

^a^Use on past 3 months.

^b^ X^2^=4.24, p=.03

^c^PANAS: Positive and Negative Affect Schedule.

^d^TM: trial making.

^e^CPT: continuous performance task, D’:
