# Supplementary material for: Brief, Web-Based Interventions to Motivate Smokers With Schizophrenia: Randomized Controlled Trial
Source: JMIR Ment Health. 2020 Feb 10;7(2):e16524. doi: 10.2196/16524 (PMC7055792; doi:10.2196/16524)
Supplement: Multimedia Appendix 2 [file mental_v7i2e16524_app2.pdf]

CONSORT-EHEALTH Checklist V1.6.2 Report	Manuscript Number	16524
**(based on CONSORT-EHEALTH V1.6), available at [http://tinyurl.com/consort-ehealth-v1-6].**		
**Date completed**		
10/7/2019 0:07:16		
**by**		
Brunette		
Randomized trial of brief, web-based interventions to motivate smokers with schizophrenia		
TITLE		
**1a-i) Identify the mode of delivery in the title**		
"Randomized trial of brief, web-based interventions to motivate smokers with schizophrenia"		
**1a-ii) Non-web-based components or important co-interventions in title**		
There are no other components		
**1a-iii) Primary condition or target group in the title**		
Yes		
ABSTRACT		
**1b-i) Key features/functionalities/components of the intervention and comparator in the METHODS section of the ABSTRACT**		
"We developed an interactive, multimedia, digital motivational decision support system for smokers with schizophrenia (Let's Talk About Smoking) that was designed to reduce cognitive load during use. We also digitalized a standard educational pamphlet from the National Cancer Institute (NCI Education) and tailored it to reduce cognitive load during use."		
**1b-ii) Level of human involvement in the METHODS section of the ABSTRACT**		
"randomly assigned receive Let's Talk about Smoking or NCI Education on a laptop computer in a single session "		
**1b-iii) Open vs. closed, web-based (self-assessment) vs. face-to-face assessments in the METHODS section of the ABSTRACT**		
"were assessed in person at 6 month follow-up. "		
**1b-iv) RESULTS section in abstract must contain use data**		
"All participants completed their assigned intervention."		
**1b-v) CONCLUSIONS/DISCUSSION in abstract for negative trials**		
Participants used their assigned interventions.		
INTRODUCTION		
**2a-i) Problem and the type of system/solution**		
"Clinics serving people with schizophrenia aim to provide interventions for schizophrenia and the common comorbidities associated with this disease. Cigarette smoking, for example, is three times more likely in people with schizophrenia than in the general population [1, 2] and leads to disparate morbidity from smoking-related diseases and early mortality [3]. However, workforce shortages are a challenge for community clinics in the U.S. and interfere with the ability to provide the array of needed interventions for smoking [4, 5]. Additionally, treatment providers experience competing demands and may lack clinical expertise for providing tobacco-related interventions [6, 7]. Deploying digital tools to deliver behavioral interventions to patients is one way to improve capacity for behavioral interventions.<br>People with schizophrenia and other serious mental illness are increasingly using digital technology, and are interested in receiving health and mental health interventions via their devices [8]. However, the design and usability of standard interventions may impede use among people in this group, who have cognitive impairments and distracting symptoms [9-12]. To address this problem, we have designed digital tools with evidence-based content that can be easily used by people with cognitive impairments and easily implemented in treatment settings where smokers with schizophrenia receive services [13, 14].<br>One potential purpose for digital tools in clinics may be to educate and motivate a user for medical treatments. A growing body of literature indicates that cessation medications with behavioral interventions are safe among people with schizophrenia [15] and increase the probability of cessation (for recent reviews, see [16-18]). However, misperceptions by both providers and patients about cessation treatment may impede utilization of these treatments [19-21]. In-person motivational and educational interventions for patients increase treatment initiation and quit attempts among smokers with severe mental illnesses [22-25]. Whether digital technology can effectively provide such interventions to people with schizophrenia has not yet been tested."		
**2a-ii) Scientific background, rationale: What is known about the (type of) system**		
"Clinics serving people with schizophrenia aim to provide interventions for schizophrenia and the common comorbidities associated with this disease. Cigarette smoking, for example, is three times more likely in people with schizophrenia than in the general population [1, 2] and leads to disparate morbidity from smoking-related diseases and early mortality [3]. However, workforce shortages are a challenge for community clinics in the U.S. and interfere with the ability to provide the array of needed interventions for smoking [4, 5]. Additionally, treatment providers experience competing demands and may lack clinical expertise for providing tobacco-related interventions [6, 7]. Deploying digital tools to deliver behavioral interventions to patients is one way to improve capacity for behavioral interventions.<br>People with schizophrenia and other serious mental illness are increasingly using digital technology, and are interested in receiving health and mental health interventions via their devices [8]. However, the design and usability of standard interventions may impede use among people in this group, who have cognitive impairments and distracting symptoms [9-12]. To address this problem, we have designed digital tools with evidence-based content that can be easily used by people with cognitive impairments and easily implemented in treatment settings where smokers with schizophrenia receive services [13, 14].<br>One potential purpose for digital tools in clinics may be to educate and motivate a user for medical treatments. A growing body of literature indicates that cessation medications with behavioral interventions are safe among people with schizophrenia [15] and increase the probability of cessation (for recent reviews, see [16-18]). However, misperceptions by both providers and patients about cessation treatment may impede utilization of these treatments [19-21]. In-person motivational and educational interventions for patients increase treatment initiation and quit attempts among smokers with severe mental illnesses [22-25]. Whether digital technology can effectively provide such interventions to people with schizophrenia has not yet been tested. "		
**Does your paper address CONSORT subitem 2b?**		
"We conducted a randomized trial of a brief, interactive, multi-media intervention (Let's Talk About Smoking) compared to a static, computerized version of an education pamphlet from the National Cancer Institute (NCI) among smokers with schizophrenia. Both interventions were tailored to reduce cognitive load on the user. We hypothesized that the rate of treatment initiation and cessation behaviors would be higher among participants assigned to Let's Talk about Smoking than among those assigned to NCI Education. Additionally, we hypothesized that level of cognitive ability would moderate participants' use of cessation treatment and ability to achieve abstinence."		
METHODS		
**3a) CONSORT: Description of trial design (such as parallel, factorial) including allocation ratio**		
"After obtaining informed consent through reading the consent form aloud and answering questions, research staff conducted baseline assessments in two, in-person meetings, with neurocognitive assessments obtained at the second meeting to reduce fatigue. Within two weeks of consent, eligible participants were randomized 1:1 to receive one of the interventions using computer-generated random order lists in blocks of eight, stratified by study site, with study participant allocation provided via pre-prepared, individual envelopes that were unsealed by research staff at the time the participant arrived for the intervention visit. Participants were not informed of the details of the study hypothesis and did not know which comparator was hypothesized to outperform the other. Using a standard protocol, research staff oriented participants to their assigned intervention, which was provided in a clinic office on a laptop computer with a mouse. They provided a brief training, coaching and assistance if needed. After completing either intervention, participants completed a computerized satisfaction questionnaire and received referral information to locally available cessation treatment (cessation medications and cessation counseling) by clinicians who were trained in providing evidence-based cessation treatment to people with serious mental illnesses. At three and six months, research interviewers assessed participants in-person for use of verifiable cessation treatment (main outcome), smoking characteristics, self-reported quit attempts (days of abstinence), and biologically verified abstinence (secondary outcomes; see Measures section), and paid participants $50."		
**3b) CONSORT: Important changes to methods after trial commencement (such as eligibility criteria), with reasons**		
No changes.		
**3b-i) Bug fixes, Downtimes, Content Changes**		
"The intervention content remained constant during the trial."		
**4a) CONSORT: Eligibility criteria for participants**		
"We enrolled English-speaking, daily smokers with schizophrenia spectrum disorders, age 18-65 years, who were psychiatrically stable in outpatient treatment for mental illness (Brief Psychiatric Rating Scale (BPRS) score <70) [26], and who were willing and able to give informed consent. Smokers were excluded if they had recently (past month) used evidence-based smoking cessation treatment (indicating the subject was already motivated to use treatment), were pregnant or nursing, or had current untreated alcohol or drug DSM-IV-TR substance dependence diagnoses. Computer experience was not required."		
**4a-i) Computer / Internet literacy**		
"Computer experience was not required."		
**4a-ii) Open vs. closed, web-based vs. face-to-face assessments:**		
"research staff conducted baseline assessments in two, in-person meetings,"		

**4a-iii) Information giving during recruitment**		
"...informed consent through reading the consent form aloud and answering questions, "		
**4b) CONSORT: Settings and locations where the data were collected**		
"Potentially eligible smokers with schizophrenia were recruited with flyers in waiting rooms and by clinician invitation from mental health treatment programs in New Jersey, Massachusetts, and Illinois in 2014-2015. "		
**4b-i) Report if outcomes were (self-)assessed through online questionnaires**		
"At three and six months, research interviewers assessed participants in-person for use of verifiable cessation treatment (main outcome), smoking characteristics, self-reported quit attempts (days of abstinence), and biologically verified abstinence..."		
**4b-ii) Report how institutional affiliations are displayed**		
"The developers and their institution were listed at the end of the intervention." AND "The publisher of the pamphlet, the National Cancer Institute, was named as sponsor of the pamphlet in standard text in the beginning and the end of the intervention."		
**5) CONSORT: Describe the interventions for each group with sufficient details to allow replication, including how and when they were actually administered**		
**5-i) Mention names, credential, affiliations of the developers, sponsors, and owners**		
"Let's Talk About Smoking is owned by the first author's primary institution"		
**5-ii) Describe the history/development process**		
Let's Talk About Smoking, is a web-based intervention tailored for smokers with severe mental illnesses and designed to increase motivation to quit smoking using evidence based treatment. The development of the intervention's content and interface involved extensive input from the intended users, and has been described previously [13]. The program is linear, modularized, and interactive, taking 30-90 minutes to complete. Users choose a video host who identifies him/ herself as an ex-smoker with mental illness and guides users through modules, each with assessments and exercises used in motivational interviewing and health decision aid systems [27, 28]. In Module 1 (Assessment/Feedback), users respond to questions and receive personalized feedback about the personal, financial and health impact of smoking. In Module 2 (Quit Intention), change decisions are facilitated by information and exercises, including creation of a personalized pros and cons list, and cessation treatment quit story videos. Module 3 (Education about cessation treatments, feedback and referral), provides selectable video quit stories as well as text and video information about cessation treatments, including the benefits of combined behavioral counseling with pharmacotherapy. A personalized report highlights desires to quit, treatment choices, and referral information. The developers and their institution were listed at the end of the intervention.<br>By developing the intervention interface and content with iterative user feedback, we ensured that the intervention was easy to use among people with the symptoms and cognitive impairments associated with psychotic disorders [13]. We previously showed that the decision support system was similarly effective among smokers with high and low levels of education, cognitive function, and symptom distress [29]. The intervention content remained constant during the trial.<br>Computerized National Cancer Institute (NCI) patient education<br>Participants assigned to NCI Education received a computerized version of the NCI patient educational handout [30], which provides information about smoking-related diseases and smoking cessation treatments. This static intervention was delivered by laptop computer in a format similar to Let's Talk About Smoking: large black font on a white background with no distracting images; one concept per page in a short paragraph or bulleted sentences. Automated audio, which read the content to users, could be turned on if the user wished. The publisher of the pamphlet, the National Cancer Institute, was named as sponsor of the pamphlet in standard text in the beginning and the end of the intervention.		
**5-iii) Revisions and updating**		
No changes were made and this is stated in the paper.		
**5-iv) Quality assurance methods**		
"Data quality was monitored throughout the study by the first author, the research data team, and a Data Safety and Monitoring Board."		
**5-v) Ensure replicability by publishing the source code, and/or providing screenshots/screen-capture video, and/or providing flowcharts of the algorithms used**		
We are not able to provide source code for the intervention.		
**5-vi) Digital preservation**		
The intervention is not available except for use in research studies.		
**5-vii) Access**		
. "Using a standard protocol, research staff oriented participants to their assigned intervention, which was provided in a clinic office on a laptop computer with a mouse. They provided a brief training, coaching and assistance if needed."		
**5-viii) Mode of delivery, features/functionalities/components of the intervention and comparator, and the theoretical framework**		
"Intervention Conditions<br>Web-based motivational intervention<br>Let's Talk About Smoking, is a web-based intervention tailored for smokers with severe mental illnesses and designed to increase motivation to quit smoking using evidence based treatment. The development of the intervention's content and interface involved extensive input from the intended users, and has been described previously [13]. The program is linear, modularized, and interactive, taking 30-90 minutes to complete. Users choose a video host who identifies him/ herself as an ex-smoker with mental illness and guides users through modules, each with assessments and exercises used in motivational interviewing and health decision aid systems [27, 28]. In Module 1 (Assessment/Feedback), users respond to questions and receive personalized feedback about the personal, financial and health impact of smoking. In Module 2 (Quit Intention), change decisions are facilitated by information and exercises, including creation of a personalized pros and cons list, and cessation treatment quit story videos. Module 3 (Education about cessation treatments, feedback and referral), provides selectable video quit stories as well as text and video information about cessation treatments, including the benefits of combined behavioral counseling with pharmacotherapy. A personalized report highlights desires to quit, treatment choices, and referral information. The developers and their institution were listed at the end of the intervention.<br>By developing the intervention interface and content with iterative user feedback, we ensured that the intervention was easy to use among people with the symptoms and cognitive impairments associated with psychotic disorders [13]. We previously showed that the decision support system was similarly effective among smokers with high and low levels of education, cognitive function, and symptom distress [29]. The intervention content remained constant during the trial.<br>Computerized National Cancer Institute (NCI) patient education<br>Participants assigned to NCI Education received a computerized version of the NCI patient educational handout [30], which provides information about smoking-related diseases and smoking cessation treatments. This static intervention was delivered by laptop computer in a format similar to Let's Talk About Smoking: large black font on a white background with no distracting images; one concept per page in a short paragraph or bulleted sentences. Automated audio, which read the content to users, could be turned on if the user wished. The publisher of the pamphlet, the National Cancer Institute, was named as sponsor of the pamphlet in standard text in the beginning and the end of the intervention."		
**5-ix) Describe use parameters**		
We specify in several places in the paper that the interventions are used in a single session.		
**5-x) Clarify the level of human involvement**		
. "Using a standard protocol, research staff oriented participants to their assigned intervention, which was provided in a clinic office on a laptop computer with a mouse. They provided a brief training, coaching and assistance if needed."		
**5-xi) Report any prompts/reminders used**		
Not necessary after the single session.		
**5-xii) Describe any co-interventions (incl. training/support)**		
. "Using a standard protocol, research staff oriented participants to their assigned intervention, which was provided in a clinic office on a laptop computer with a mouse. They provided a brief training, coaching and assistance if needed."		
**6a) CONSORT: Completely defined pre-specified primary and secondary outcome measures, including how and when they were assessed**		
Primary outcome – Confirmed use of smoking cessation treatment and quit attempts Blinded assessors completed a structured interview to assess all self-reported use of cessation treatment (including nicotine replacement therapy) at any time during each past three month period. Use of cessation treatment was confirmed via clinic record review, clinician confirmation, and viewing medications and nicotine replacement at the assessment.<br>Secondary outcome – Abstinence At the follow-up assessment visits, self-reported past week of abstinence from all tobacco products was verified with expired carbon monoxide (CO) less than 9 ppm (Smokelyzer Breath Carbon Monoxide Monitor, Bedfont Scientific) [36, 37]. Additionally, any self-reported quit attempts with abstinence during the treatment period were captured with the Timeline Follow-Back method [38-40]. With this method, trained research staff assessed subjects for amount of smoking and other tobacco product use each day, going back week-by-week over the past three months using a calendar to cue memories of smoking and abstinence. The Timeline Follow-Back method has been shown to be reliable and valid in the general population [40] and in people with severe mental illnesses [41].<br>Intervention satisfaction, usability and likeability Participants completed the Perceived Usefulness and Ease of Use Scale, an adapted 15-item semi-qualitative instrument [42] to obtain perceptions of usability and satisfaction with the intervention.		
**6a-i) Online questionnaires: describe if they were validated for online use and apply CHERRIES items to describe how the questionnaires were designed/deployed**		
No online questionnaires.		
**6a-ii) Describe whether and how "use" (including intensity of use/dosage) was defined/measured/monitored**		
"Using a standard protocol, research staff oriented participants to their assigned intervention, which was provided in a clinic office on a laptop computer with a mouse. They provided a brief training, coaching and assistance if needed."		
**6a-iii) Describe whether, how, and when qualitative feedback from participants was obtained**		
"participants completed a computerized satisfaction questionnaire (computerized to reduce response bias), "		

**6b) CONSORT: Any changes to trial outcomes after the trial commenced, with reasons**		
"Potentially eligible smokers with schizophrenia were recruited with flyers in waiting rooms and by clinician invitation from mental health treatment programs in New Jersey, Massachusetts, and Illinois in 2014-2015. "		
**7a) CONSORT: How sample size was determined**		
**7a-i) Describe whether and how expected attrition was taken into account when calculating the sample size**		
We did take attrition into account in calculating sample size.		
**7b) CONSORT: When applicable, explanation of any interim analyses and stopping guidelines**		
Primary outcome – Confirmed use of smoking cessation treatment and quit attempts Blinded assessors completed a structured interview to assess all self-reported use of cessation treatment (including nicotine replacement therapy) at any time during each past three month period. Use of cessation treatment was confirmed via clinic record review, clinician confirmation, and viewing medications and nicotine replacement at the assessment.<br>Secondary outcome – Abstinence At the follow-up assessment visits, self-reported past week of abstinence from all tobacco products was verified with expired carbon monoxide (CO) less than 9 ppm (Smokelyzer Breath Carbon Monoxide Monitor, Bedfont Scientific) [36, 37]. Additionally, any self-reported quit attempts with abstinence during the treatment period were captured with the Timeline Follow-Back method [38-40]. With this method, trained research staff assessed subjects for amount of smoking and other tobacco product use each day, going back week-by-week over the past three months using a calendar to cue memories of smoking and abstinence. The Timeline Follow-Back method has been shown to be reliable and valid in the general population [40] and in people with severe mental illnesses [41].<br>Intervention satisfaction, usability and likeability Participants completed the Perceived Usefulness and Ease of Use Scale, an adapted 15-item semi-qualitative instrument [42] to obtain perceptions of usability and satisfaction with the intervention.		
**8a) CONSORT: Method used to generate the random allocation sequence**		
"Within two weeks of consent, eligible participants were randomized 1:1 to receive one of the interventions using computer-generated random order lists in blocks of eight, stratified by study site, with study participant allocation provided via pre-prepared, individual envelopes that were unsealed by research staff at the time the participant arrived for the intervention visit."		
**8b) CONSORT: Type of randomisation; details of any restriction (such as blocking and block size)**		
"Within two weeks of consent, eligible participants were randomized 1:1 to receive one of the interventions using computer-generated random order lists in blocks of eight, stratified by study site, with study participant allocation provided via pre-prepared, individual envelopes that were unsealed by research staff at the time the participant arrived for the intervention visit."		
**9) CONSORT: Mechanism used to implement the random allocation sequence (such as sequentially numbered containers), describing any steps taken to conceal the sequence until interventions were assigned**		
"Within two weeks of consent, eligible participants were randomized 1:1 to receive one of the interventions using computer-generated random order lists in blocks of eight, stratified by study site, with study participant allocation provided via pre-prepared, individual envelopes that were unsealed by research staff at the time the participant arrived for the intervention visit. "		
**10) CONSORT: Who generated the random allocation sequence, who enrolled participants, and who assigned participants to interventions**		
"Within two weeks of consent, eligible participants were randomized 1:1 to receive one of the interventions using computer-generated random order lists in blocks of eight, stratified by study site, with study participant allocation provided via pre-prepared, individual envelopes that were unsealed by research staff at the time the participant arrived for the intervention visit. "		
**11a) CONSORT: Blinding - If done, who was blinded after assignment to interventions (for example, participants, care providers, those assessing outcomes) and how**		
**11a-i) Specify who was blinded, and who wasn't**		
"At three and six months, blinded research interviewers assessed participants in-person for use of verifiable cessation treatment (main outcome), smoking characteristics, self-reported quit attempts (days of abstinence), and biologically verified abstinence (secondary outcomes; see Measures section), "		
**11a-ii) Discuss e.g., whether participants knew which intervention was the "intervention of interest" and which one was the "comparator"**		
"Participants were not informed of the details of the study hypothesis and did not know which comparator was hypothesized to outperform the other. "		
**11b) CONSORT: If relevant, description of the similarity of interventions**		
The interventions were described in detail as shown in quotes above.		
**12a) CONSORT: Statistical methods used to compare groups for primary and secondary outcomes**		
"Statistical analyses<br>We used chi-squared tests and t-tests to assess between-group differences at baseline. We then assessed dichotomous outcomes between intervention groups with logistic regressions (e.g. treatment use) [53]. For count outcome variables with a high proportion of zeros and positive skewness (e.g., biologically verified point prevalence abstinence), negative binomial models were used. Modeling began with bivariates and progressed to multivariates using variables providing p< .10 in bivariate models, adjusting for gender and years of education. In the multivariate model predicting any abstinence, the total mean cognitive battery score was utilized to avoid collinearity among the cognitive function scores. Missing observations for the primary outcome, cessation treatment utilization were set as missing. Missing observations for the secondary outcome, abstinence, were set as smoking (nonabstinent). Analyses were conducted with SAS Version 9.4 (SAS Institute, Cary, N.C.)."		
**12a-i) Imputation techniques to deal with attrition / missing values**		
" Missing observations for the primary outcome, cessation treatment utilization were set as missing. Missing observations for the secondary outcome, abstinence, were set as smoking (nonabstinent)."		
**12b) CONSORT: Methods for additional analyses, such as subgroup analyses and adjusted analyses**		
"Statistical analyses<br>We used chi-squared tests and t-tests to assess between-group differences at baseline. We then assessed dichotomous outcomes between intervention groups with logistic regressions (e.g. treatment use) [53]. For count outcome variables with a high proportion of zeros and positive skewness (e.g., biologically verified point prevalence abstinence), negative binomial models were used. Modeling began with bivariates and progressed to multivariates using variables providing p< .10 in bivariate models, adjusting for gender and years of education. In the multivariate model predicting any abstinence, the total mean cognitive battery score was utilized to avoid collinearity among the cognitive function scores. Missing observations for the primary outcome, cessation treatment utilization were set as missing. Missing observations for the secondary outcome, abstinence, were set as smoking (nonabstinent). Analyses were conducted with SAS Version 9.4 (SAS Institute, Cary, N.C.)."		
RESULTS		
**13a) CONSORT: For each group, the numbers of participants who were randomly assigned, received intended treatment, and were analysed for the primary outcome**		
Shown in Figure 1, consort diagram for subject flow. . "In total, 184 subjects were consented and assessed for eligibility; 173 were eligible, 162 were randomized and received study interventions, and 145 (89.5% of those randomized) completed the six-month follow-up (see Figure 1 for participant flow)."		
**13b) CONSORT: For each group, losses and exclusions after randomisation, together with reasons**		
Also shown in Figure 1. ". In total, 184 subjects were consented and assessed for eligibility; 173 were eligible, 162 were randomized and received study interventions, and 145 (89.5% of those randomized) completed the six-month follow-up (see Figure 1 for participant flow)."		
**13b-i) Attrition diagram**		
. "In total, 184 subjects were consented and assessed for eligibility; 173 were eligible, 162 were randomized and received study interventions, and 145 (89.5% of those randomized) completed the six-month follow-up (see Figure 1 for participant flow)." AND " Intervention Usability and Satisfaction Usability and satisfaction mean summary index scores were significantly higher among participants assigned to Let's Talk About Smoking compared to those assigned to NCI education (8.9±1.3 vs. 8.3±2.1, df= 120.7, t= 2.0, p=.045). All participants completed the intervention to which they were assigned; no adverse events were reported during the use of the interventions. About 97% of both groups said they would recommend their respective intervention to a friend."		
**14a) CONSORT: Dates defining the periods of recruitment and follow-up**		
"Potentially eligible smokers with schizophrenia were recruited with flyers in waiting rooms and by clinician invitation from mental health treatment programs in New Jersey, Massachusetts, and Illinois in 2014-2015. "		
**14a-i) Indicate if critical "secular events" fell into the study period**		
No known secular events.		
**14b) CONSORT: Why the trial ended or was stopped (early)**		
Not applicable.		
**15) CONSORT: A table showing baseline demographic and clinical characteristics for each group**		
Table 1 shown these characteristics.		
**15-i) Report demographics associated with digital divide issues**		
Yes		
**16a) CONSORT: For each group, number of participants (denominator) included in each analysis and whether the analysis was by original assigned groups**		
**16-i) Report multiple "denominators" and provide definitions**		
No relevant for this study - all 162 received interventions.		
**16-ii) Primary analysis should be intent-to-treat**		

All participants received intervention.		
**17a) CONSORT: For each primary and secondary outcome, results for each group, and the estimated effect size and its precision (such as 95% confidence interval)**		
"...cessation treatment use was not different between intervention groups (32.1% of Let's Talk About Smoking vs. 46.2% NCI Education; OR = 0.71 [0.37-1.33]; p=0.28). "		
**17a-i) Presentation of process outcomes such as metrics of use and intensity of use**		
"All participants completed the intervention to which they were assigned; no adverse events were reported during the use of the interventions."		
**17b) CONSORT: For binary outcomes, presentation of both absolute and relative effect sizes is recommended**		
"As shown in Table 2, over a third (n=63, 38.9%) of all participants utilized any verifiable cessation treatment during the six-month follow-up period, and cessation treatment use was not different between intervention groups (32.1% of Let's Talk About Smoking vs. 46.2% NCI Education; OR = 0.71 [0.37-1.33]; p=0.28). Twenty-one participants (13.0%) had used any verified cessation medication, 21 (13.0%) had used any verified behavioral intervention, and the same number had used the recommended combination of both a behavioral and a medication intervention (n=21, 13.0%). A larger number of participants self-reported use of treatment or had verified use of treatment (also shown in Table 2). In bivariate logistic models, any treatment initiation was significantly predicted by older age (OR = 1.03 [1.00-1.06], p=.05), higher levels of education (OR=1.18 [1.02, 1.37], p=.02), and lower positive symptom scale scores (OR=.87 [0.79-0.95), p=.00). In the full multivariate model predicting cessation treatment utilization, older age, higher education, and lower level of positive symptoms scores remained significant predictors of treatment initiation (See Table 3)."		
**18) CONSORT: Results of any other analyses performed, including subgroup analyses and adjusted analyses, distinguishing pre-specified from exploratory**		
Not applicable		
**18-i) Subgroup analysis of comparing only users**		
Not applicable		
**19) CONSORT: All important harms or unintended effects in each group**		
"Intervention Usability and Satisfaction<br>Usability and satisfaction mean summary index scores were significantly higher among participants assigned to Let's Talk About Smoking compared to those assigned to NCI education (8.9±1.3 vs. 8.3±2.1, df= 120.7, t= 2.0, p=.045). All participants completed the intervention to which they were assigned; no adverse events were reported during the use of the interventions. About 97% of both groups said they would recommend their respective intervention to a friend. "		
**19-i) Include privacy breaches, technical problems**		
Not relevant		
**19-ii) Include qualitative feedback from participants or observations from staff/researchers**		
"Intervention Usability and Satisfaction<br>Usability and satisfaction mean summary index scores were significantly higher among participants assigned to Let's Talk About Smoking compared to those assigned to NCI education (8.9±1.3 vs. 8.3±2.1, df= 120.7, t= 2.0, p=.045). All participants completed the intervention to which they were assigned; no adverse events were reported during the use of the interventions. About 97% of both groups said they would recommend their respective intervention to a friend. "		
DISCUSSION		
**20) CONSORT: Trial limitations, addressing sources of potential bias, imprecision, multiplicity of analyses**		
**20-i) Typical limitations in ehealth trials**		
"Several study limitations should be mentioned. First, we were not able to obtain detailed information about the frequency and intensity of the community-delivered cessation medication and behavioral interventions, which would have facilitated better understand our secondary abstinence outcome. Additionally, this study utilized an active, computerized control condition, thus we were unable to determine the level of advantage these interventions provide over usual care, such as doctor's advice. Study participants were recruited from three large community clinics in three states and included smokers with schizophrenia from several racial and ethnic groups, yet they may not be representative of all smokers with schizophrenia in the U.S. or other countries."		
**21) CONSORT: Generalisability (external validity, applicability) of the trial findings**		
**21-i) Generalizability to other populations**		
"Study participants were recruited from three large community clinics in three states and included smokers with schizophrenia from several racial and ethnic groups, yet they may not be representative of all smokers with schizophrenia in the U.S. or other countries."		
**21-ii) Discuss if there were elements in the RCT that would be different in a routine application setting**		
Not applicable - we used routine clinical settings		
**22) CONSORT: Interpretation consistent with results, balancing benefits and harms, and considering other relevant evidence**		
**22-i) Restate study questions and summarize the answers suggested by the data, starting with primary outcomes and process outcomes (use)**		
DISCUSSION<br>Contrary to our hypothesis, smokers with schizophrenia assigned to the interactive intervention were not more likely to initiate cessation treatment. Yet these brief, digital interventions led to rates of treatment engagement consistent with studies of earlier versions of Let's Talk About Smoking [54-56] and consistent with in-person motivational interviewing, in which 28-32.7% of smokers with schizophrenia and bipolar disorder attended an initial treatment appointment [22, 25]. This study suggests that carefully designed, automated, digital interventions could be used to engage this population into quit attempts using evidence-based smoking cessation treatment. The interactive, multimedia intervention was significantly more appealing than the static educational intervention, indicating that future uptake of digital interventions in a non-study environment could be more successful with an interactive, multimedia approach.		
**22-ii) Highlight unanswered new questions, suggest future research**		
Further research is warranted to evaluate efficacy and implementation strategies for digital interventions for smokers with schizophrenia and other serious mental illnesses.		
Other information		
**23) CONSORT: Registration number and name of trial registry**		
Yes		
**24) CONSORT: Where the full trial protocol can be accessed, if available**		
Not available.		
**25) CONSORT: Sources of funding and other support (such as supply of drugs), role of funders**		
NCI was only funder - "Funding: This work was supported by the National Cancer Institute (grant #1R01CA168778-01A1 REVISED)."		
**X26-i) Comment on ethics committee approval**		
Data quality was monitored throughout the study by the first author, the research data team, and a Data Safety and Monitoring Board. The study was reviewed and monitored by the Dartmouth Committee for the Protection of Human Subjects and the Institutional Review Boards of research sites.		
**x26-ii) Outline informed consent procedures**		
"After obtaining informed consent through reading the consent form aloud and answering questions, research staff conducted baseline assessments ..."		
**X26-iii) Safety and security procedures**		
There were no safety issues.		
**X27-i) State the relation of the study team towards the system being evaluated**		
"Conflict of Interest: During the study period, Dr. (FILL IN) had funding from Alkermes to conduct research on medication treatment for schizophrenia and alcohol disorder. The remaining authors did not report potential conflicts of interest. Let's Talk About Smoking is owned by the first author's primary institution."		
